# Supplementary material for: Physical limits to biomechanical sensing in disordered fibre networks
Source: Nat Commun. 2017 Jul 18;8:16096. doi: 10.1038/ncomms16096 (PMC5520107; doi:10.1038/ncomms16096)
Supplement: Supplementary Information [file ncomms16096-s1.pdf]

SI GUIDE

Type of file: pdf
Size of file: 13,008 KB
Title of file for HTML: Supplementary Information
Description: Supplementary Figures, Supplementary Tables, Supplementary Methods and Supplementary References.

Type of file: pdf
Size of file: 364 KB
Title of file for HTML: Peer Review File
Description:

Supplementary Figures

Analysis of local stiffness

Experimental network architectures

For each experimental network, the corresponding mechanical model has two undetermined parameters: the stretching modulus μ and the bending modulus κ. Since both parameters may be rescaled together resulting only in a trivial change of units, the only remaining non-trivial parameter is their ratio κ/μ. Throughout the main text, we chose $\kappa/\mu = 10^{-5}$ to capture the fact that individual fibers are much stiffer to stretching than to bending.

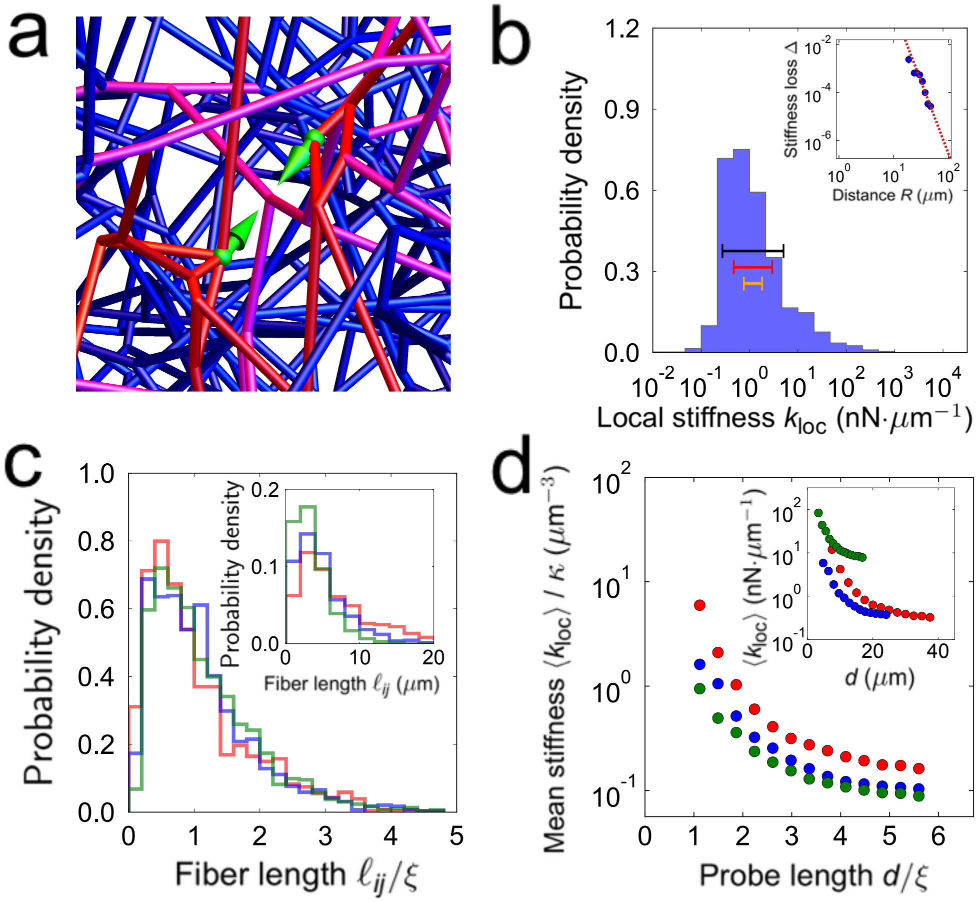

Supplementary Figure 1. Analysis of experimental fibrin network. (**a**) Modeled deformation under stress from a local force dipole of length $d \sim 15\mu$m (green arrows) of experimental fibrin network ($c = 0.2$ mg ml^{-1}). Magnitude of fiber deformations indicated by color (small deformations blue, large deformations red). (**b**) Distribution of local stiffnesses $k_{\rm loc}$ (force dipole of length $d \sim 15\mu$m) for experimental fibrin network ($c = 0.2$ mg ml^{-1}). Geometric standard deviation of local stiffness $\sigma_{\rm loc}$ indicated by bars (actual distribution black, estimated distribution assuming strong locality red, estimated distribution assuming weak locality orange. Inset shows stiffness loss Δ, defined as the relative change in local stiffness $k_{\rm loc}$ upon perturbing a network by removing a single fiber, versus distance R of center of removed fiber from probe center (probe length $d < 10\mu$m and removed-fiber length $\ell_{ij} < 10\mu$m). Error bars, defined as the standard deviation of each data point divided by the square root of the number of samples averaged, are smaller than the size of data points. Dashed lines show asymptotic scaling from continuum theory, which predicts $\Delta \sim 1/R^{2D}$ for $R >> d$. (**c**) Distribution of rescaled fiber lengths ℓ_{ij}/ξ, defined as the fiber length ℓ_{ij} divided by mesh size ξ, for experimental fibrin networks of varying concentration ($c = 0.2$ mg ml^{-1} red, $c = 0.8$ mg ml^{-1} blue, $c = 1.6$ mg ml^{-1} green). Inset shows distributions of fiber lengths ℓ_{ij}. (**d**) Rescaled geometric mean of local stiffness $\langle k_{\rm loc}\rangle/\kappa$, defined as the geometric mean of local stiffness $\langle k_{\rm loc}\rangle$ divided by the bending modulus κ, versus rescaled probe length d/ξ, defined as the probe length d divided by the mesh size ξ for experimental fibrin networks of varying concentration ($c = 0.2$ mg ml^{-1} red, $c = 0.8$ mg ml^{-1} blue, $c = 1.6$ mg ml^{-1} green). Inset shows geometric mean of local stiffness $\langle k_{\rm loc}\rangle$ versus probe length d.

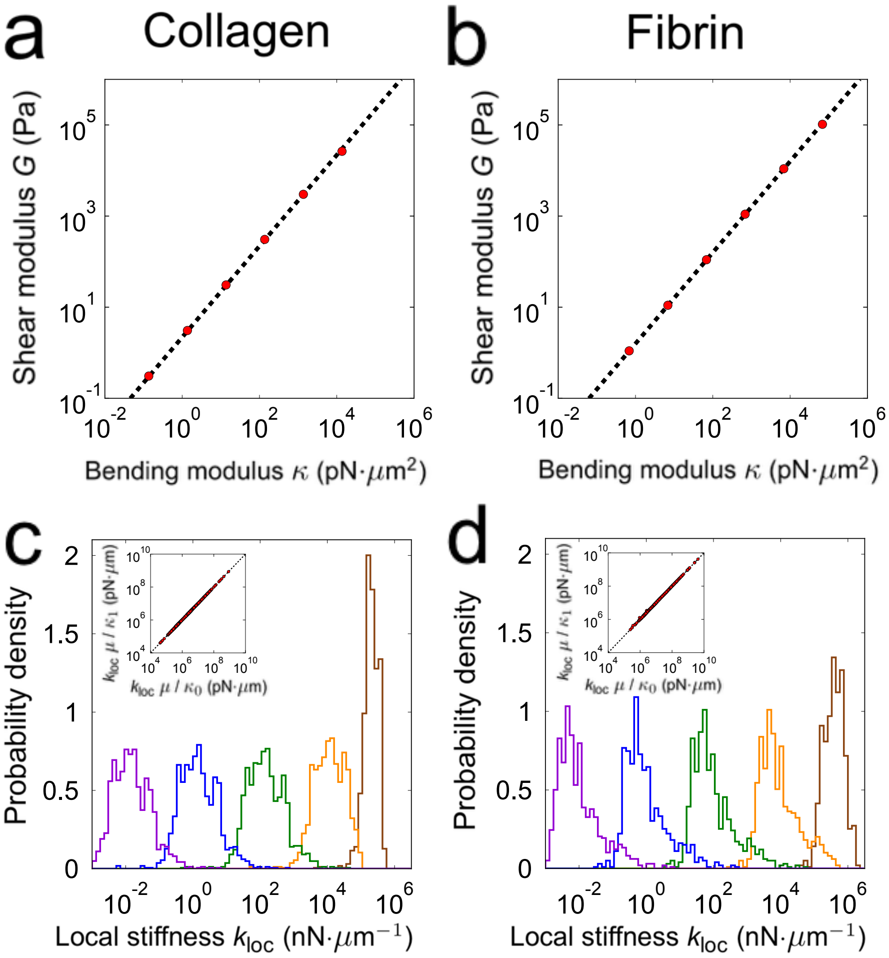

Supplementary Figure 2. Analysis of experimental networks. (**a**,**b**) Macroscopic shear modulus G versus bending modulus κ for (**a**) experimental collagen network and (**b**) experimental fibrin network. (**c**,**d**) Distribution of local stiffnesses k_{loc} for a broad range of bending moduli for (**c**) experimental collagen network (κ is set to κ_0 purple, $10^2\kappa_0$ blue, $10^4\kappa_0$ green, $10^6\kappa_0$ orange, and $10^8\kappa_0$ brown, where $\kappa_0 = 0.4$ nN·μm^2) and (**d**) experimental fibrin network (κ is set to κ_0 purple, $10^2\kappa_0$ blue, $10^4\kappa_0$ green, $10^6\kappa_0$ orange, and $10^8\kappa_0$ brown, where $\kappa_0 = 2$ nN·μm^2). Insets show comparison of stiffness measurements for two different values of bending modulus, κ_0 and $10^2\kappa_0$, scaled by bending modulus for (**c**) collagen network ($\kappa_0 = 0.4$ nN·μm^2) and (**d**) fibrin network ($\kappa_0 = 2$ nN·μm^2). Dashed line shows linear fit.

We compute the local stiffness distribution numerically using the procedure described in the Methods section of the main text. However, rather than considering an ensemble of individual probes over many network realizations, we sample many probes throughout the volume of a single network. These procedures are equivalent when the sample volumes are large compared to the scale over which local stiffness measurements are correlated, i.e. networks which are large enough to represent the ensemble via self-averaging. We confirmed this equivalence by varying the sample volume and observing that the distribution converged below the network radius used in our analysis.

To determine the units of the modeled response, we first noted that both the experimental collagen and fibrin networks display a bending-dominated response. That is, for these networks, the macroscopic shear modulus and all local stiffness measurements are directly proportional to κ (Supplementary Fig. 2a-d). We therefore fit the bending modulus of our mechanical model using the experimentally-measured macroscopic response. Specifically, we first calculated the shear modulus numerically using the mechanical model for an arbitrary value of κ, which we then scaled by the ratio of the experimental shear modulus to the calculated shear modulus. This resulted in fitted bending moduli of $\kappa = 0.38$, 2.0, 3.6, and 90 nN·μm^2 for collagen at $c = 0.2$ and fibrin at $c = 0.2$, $c = 0.8$, and $c = 1.6$ mg ml^{-1}, respectively.

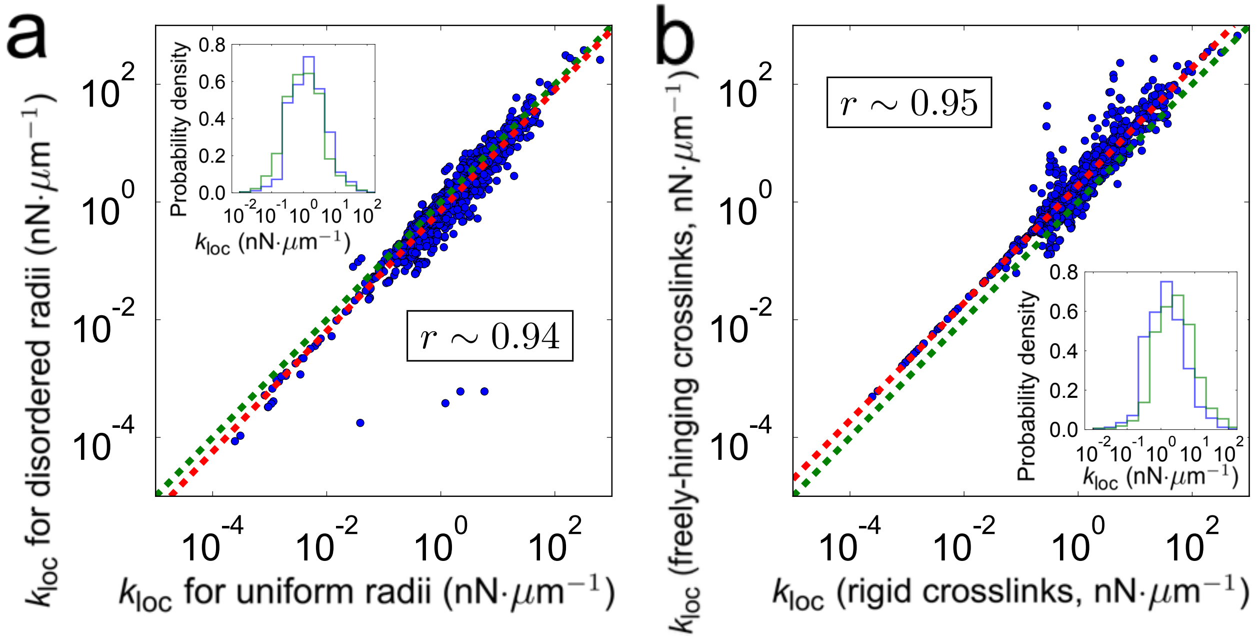

Supplementary Figure 3. Variations of the mechanical model. (**a**) Case-by-case comparison of modeled local stiffnesses for experimental collagen network (force dipole of length $d \sim 15\mu$m) modeled with fiber radii randomly distributed according to a Gaussian distribution ($\langle a \rangle = 83$ nm and $\sigma(a) = 50$ nm) versus same network modeled with uniform fiber radii ($a = 83$ nm). Green line indicates identity function. Red line indicates best linear fit of log-local stiffnesses (correlation coefficient $r = 0.94$). Inset shows local stiffness distributions (force dipole length $d = 15$ μm) for each model (uniform radii blue, disordered radii green). (**b**) Case-by-case comparison of modeled local stiffnesses for experimental collagen network (force dipole of length $d \sim 15\mu$m) modeled with freely-hinging crosslinks at $z = 4$ coordinated vertices versus same network modeled with rigid junctions at $z = 4$ coordinated vertices. Green line indicates identity function. Red line indicates best linear fit of log-local stiffnesses (correlation coefficient $r = 0.95$). Inset shows local stiffness distributions (force dipole length $d = 15$ μm) for each model (rigid crosslinks blue, freely-hinging crosslinks green).

Local stiffnesses are robust to fiber thickness disorder

A major simplification of our model was to assume that all fibers throughout the network have identical mechanical properties. However, both experimentally reconstituted networks *in vitro* and biological fiber networks *in vivo* contain fibers of varying thickness. For example, collagen fibers are known to range from 25 nm to 100 nm in radius[1], and fibrin fibers are known to range from 25 nm to 100 nm in diameter[2]. How would the presence of variation in fiber radius impact our results? The bending modulus of a homogeneous rod is given by[3]:

$$\kappa = \frac{\pi}{4} E a^4, \tag{1}$$

where E is the Young's modulus of the fiber and a is its radius. Given this sensitive dependence of the bending modulus on fiber radius, we would like to know whether our simplified mechanical model with fixed κ can provide accurate estimates of local stiffness.

To determine how our results depend on the additional disorder due to varying fiber thickness, we extended our mechanical model to allow for a distribution of bending moduli:

$$H_\kappa = \frac{\mu}{2} \sum_{\langle ij \rangle} \frac{1}{\ell_{ij}} \left(\boldsymbol{u}_{ij} \cdot \hat{\boldsymbol{r}}_{ij}\right)^2 + \frac{1}{2} \sum_{\langle ijk \rangle} \frac{\kappa_{ijk}}{\ell_{ijk}} \left(\frac{\boldsymbol{u}_{jk} \times \hat{\boldsymbol{r}}_{ij}}{\ell_{jk}} - \frac{\boldsymbol{u}_{ij} \times \hat{\boldsymbol{r}}_{jk}}{\ell_{ij}} \right)^2, \tag{2}$$

where κ_{ijk} is the bending modulus for the bending interaction of fibers ij and jk. Since the resolution on our confocal images (200 to 500 nm) was too low to extract the fiber radii, we considered a random distribution of modeled bending moduli. Specifically, we first assigned a radius a_i to each fiber segment by sampling from a random distribution of fiber radii. For simplicity, we chose the fiber radii to be normally distributed with a mean $\langle a \rangle$ equal to the inferred

value of fiber radius ($\langle a \rangle = 83$ nm, obtained by fitting the bending modulus to macroscopic rheology and assuming fibers each have a uniform radius and a Young's modulus of 10 MPa), and a standard deviation $\sigma_a = 50$ nm to mimic the experimentally observed variability[1].

Thus, each fiber segment was associated with a bending rigidity $\kappa_{ij} \sim a_{ij}^4$. Two connected fiber segments of different radii form a composite rod, whose bending interaction can be described by a single effective modulus. How would this effective modulus depend on the bending moduli and lengths of the segments? For the case of two identical segments, the bending interaction is treated as a torsional spring with an angular spring constant given by κ_{ij}/ℓ_{ij}, where ℓ_{ij} is the length of the segment. However, when the lengths and radii of the segments differ, the interaction modeled by the torsional spring corresponds to deformations and curvatures that are distributed nonuniformly over the two segments. The full energy for the corresponding worm-like chain (WLC) Hamiltonian has a complicated dependence on the angle between the segments[4]. Here, we approximate the effective bending modulus of two connected fiber segments ij and jk as the harmonic mean of their "spring constants" κ_{ij}/ℓ_{ij} and κ_{jk}/ℓ_{jk} as follows:

$$\kappa_{ijk} = 2\frac{\kappa_{ij}\kappa_{jk}}{\kappa_{ij}\ell_{jk} + \kappa_{jk}\ell_{ij}}. \tag{3}$$

In the limit that the bending moduli of the fibers are equal, the above expression reduces to our initial simplified model, which has previously been shown to provide a good approximation to the full energy given by the WLC model[5].

For the spatially disordered collagen network, we generated a random distribution of fiber radii and compared the resulting modeled local stiffnesses to those obtained using our original model with uniform bending moduli. This allowed us to determine the effect of heterogeneity in fiber radii while controlling for the effect of network geometry. Remarkably, the modeled values of local stiffness are relatively unchanged by introducing a distribution of fiber radii (Supplementary Fig. 3a). This lack of relative change in the modeled local stiffnesses is reflected in the extremely high $r \sim 0.94$ correlation between log-local stiffnesses measured for the uniform radii model versus the non-uniform radii model. Consequently, the presence of a broad distribution of local stiffnesses is robust to the additional mechanical disorder due to fiber radii, with only a slight increase in the geometric standard deviation from $\sigma_{\text{loc}} = 0.63$ to $\sigma_{\text{loc}} = 0.69$ (inset of Supplementary Fig. 3a). This indicates that the effect of spatial disorder due to the randomly positioned vertices and fibers dominates over the effect of mechanical disorder due to varying fiber thickness.

Local stiffnesses are robust to junction mechanics model

Biopolymers can be assembled into networks in a variety of different ways. In particular, multiple constituent fibers may be crosslinked together by a diverse collection of accessory proteins[6], or they can form bundles that branch off at specific angles[7]. The former typically corresponds to a coordination number of $z = 4$ (two fibers joined at a point), and the latter to $z = 3$ (a single fiber that splits into two). We expect these varying interactions to occur at the junctions of our reconstituted networks, which contain vertices that are primarily three-fold and four-fold coordinated. However, our experimental approach cannot distinguish crosslinking from branching. In our mechanical model, for simplicity we have chosen to treat all junctions equally. That is, we included bending interactions over all connected pairs of fiber segments, and we assumed that the form of the interaction is the same for all such pairs. How sensitively do our results depend on the type of interactions occurring at the junctions?

To check whether our modeled local stiffnesses depend strongly on the type of interactions at fiber junctions, we considered an alternative model that includes freely-hinging junctions. Specifically, to model the effect of crosslinking, we considered a single freely-hinging junction at each $z = 4$ coordinated vertex, rather than including a bending interaction constraint over all pairs of fibers at each junction. Since we could not directly observe whether connected fiber segments belong to the same fiber, we randomly assigned two pairs of fiber segments at $z = 4$ vertices to belong to the same fiber. For the freely-hinging crosslink model, only fiber segments belonging to the same fiber contributed an angle-constraining bending interaction at each junction, whereas for the rigid crosslink model, all connected pairs of fiber segments contributed an angle-constraining bending interaction.

We compared the freely-hinging crosslink mechanical model to our rigid crosslink mechanical model for the collagen network. We found that not only is the broad width of the distribution robust to the choice of junction interactions, but furthermore, our modeled local stiffnesses were remarkably unchanged on a case-by-case basis (Supplementary Fig. 3b). This suggests that the dominant contribution to the disorder in local stiffness comes from the intrinsic structural disorder of the network, rather than the details of the interaction topology at junctions. Therefore, our fiber network model should provide reliable estimates for local stiffness even in the absence of detailed knowledge of the interactions occurring at junctions.

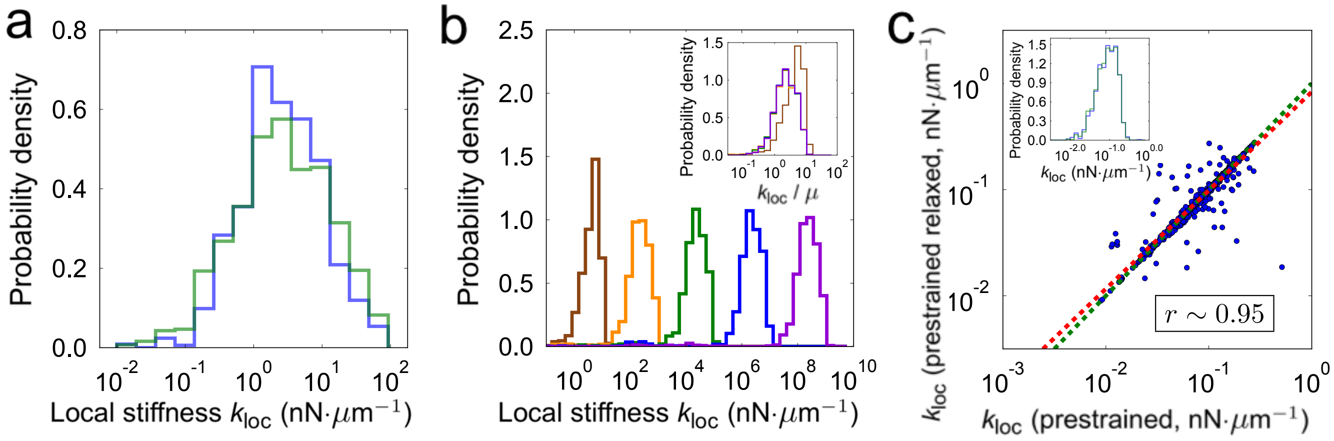

Supplementary Figure 4. Analysis of collagen network architecture using nonlinear Hamiltonian. (**a**) Distribution of local stiffnesses $k_{\rm loc}$ (force dipole of length $d \sim 10\mu$m) for experimental collagen network for linear Hamiltonian $H_{\rm lin}$ (blue) and nonlinear Hamiltonian H for small deformations (green). (**b**) Distribution of local stiffnesses $k_{\rm loc}$ (force dipole of length $d \sim 10\mu$m, macroscopic strain $\gamma = 0.2$) for a broad range of stretching moduli for experimental collagen network (μ is set to μ_0 brown, $10^2\mu_0$ yellow, $10^4\mu_0$ green, $10^6\mu_0$ blue, and $10^8\mu_0$ purple, where $\mu_0 = 0.4$ nN μm^{-1}) for fixed value of the bending modulus $\kappa = 0.4$ nN·μm^2. Inset shows distribution of scaled local stiffnesses $k_{\rm loc}/\mu$, defined as the local stiffness $k_{\rm loc}$ divided by the stretching modulus μ (force dipole of length $d \sim 10\mu$m), for a broad range of stretching moduli for experimental collagen network (for the same set of μ and choice of κ as in the main panel). (**c**) Case-by-case comparison of modeled local stiffnesses for experimental collagen network (force dipole of length $d \sim 10\mu$m) for the prestrained network state versus the prestrained relaxed network state. Green line indicates identity function. Red line indicates best linear fit of log-local stiffnesses (correlation coefficient $r = 0.95$). Inset shows local stiffness distributions (force dipole length $d = 10$ μm) for each network state (prestrained blue, prestrained relaxed green).

Nonlinear response of collagen network

We find that for small deformations, our results do not depend significantly on whether we use the linear or the nonlinear Hamiltonian (Supplementary Fig. 4a). Therefore, throughout our analysis we typically use the linear Hamiltonian, with the exception of when we study the effect of network preexisting strain.

To study how the local stiffness would be affected by the presence of forces that nonlinearly deform the network, we considered the local stiffness distribution for networks with a preexisting strain, or "prestrain." For simplicity, we considered prestrains consisting of macroscopically uniform dilations. We numerically modeled such dilations by first applying a uniform, affine dilation to a network, next pinning the vertices at a buffer region near the boundary, and finally allowing the network to relax using a gradient-descent algorithm. We then obtain the local stiffness as before, however here the dynamical matrix is given by the second derivative of the nonlinear Hamiltonian with respect to the positions of the vertices.

In the main text, we found that such prestrains can cause initially bending-dominated networks to yield bimodal distributions of local stiffnesses (Fig. 4). The second peak consists of local stiffnesses that are significantly stiffer than those observed in the initial, bending-dominated network. Furthermore, the width of the second peak is slightly narrower than the width of the local stiffness distribution for the undeformed network. Both of these features are reminiscent of the crossover in the local stiffness distribution as connectivity is increased until the network transitions from a bending-dominated elastic regime to a stretching-dominated elastic regime. To determine the mechanical regime of the second peak, we systematically varied the stretching modulus for a fixed value of the bending modulus for the experimental collagen network under large modeled strain (force dipole length $d = 15$ μm, network strain $\gamma = 0.2$). For this prestressed network, we found that the entire local stiffness distribution scales with the stretching modulus (Supplementary Fig. 4b), which suggests that prestress can induce an elastic crossover.

Local stiffnesses are robust to plastic deformations

The mechanical response of collagen and fibrin networks is known to change in response to repeated, large-strain loading[23,51]. This dynamic evolution was found to be caused by persistent lengthening of individual fibers, rather than by the rupture of individual fibers or detachment of branch points[51]. We have neglected this plastic deformation

in our analysis of local stiffness by treating fibers as elastic elements. How strongly do our results for the local stiffness distribution depend on this assumption of our model?

For fibers that undergo persistent lengthening upon loading, successive stiffness measurements applied to the same location may yield different values of local stiffness. Based on the observed dynamical evolution of the mechanical response[51], this fiber lengthening effect should be relevant for networks that have been deformed enough to enter the nonlinear strain-stiffening regime. We therefore employ our model for the nonlinear response in conjunction with irreversible fiber lengthening to study the effect of repeated, large-strain loading on the local stiffness. We begin by numerically simulating the effect of a large prestrain on the network as described above. To be concrete, we choose $\gamma = 0.5$ to ensure that the network is well beyond the bending-to-stretching crossover and therefore in the relevant regime for substantial plastic deformations. We refer to this network state as the "prestrained" state. We then model persistent lengthening of fibers by applying an irreversible lengthening to any fibers that have been stretched longer than their length ℓ_{ij} in the undeformed reference state. Specifically, we perform this lengthening by taking $\ell_{ij} \to \ell^*_{ij}$ in our nonlinear Hamiltonian, where ℓ^*_{ij} is the length of the fiber ij in the prestrained state. Then we allow the network to relax using a gradient-descent algorithm and the same boundary conditions as for the prestrained state. We refer to the resulting state as the "prestrained relaxed" state. Finally, we obtain the local stiffnesses as described above.

By comparing the local stiffnesses for the prestrained state to those of the prestrained relaxed state, we found that our modeled local stiffnesses were remarkably unchanged on a case-by-case basis (Supplementary Fig. 4c). The negligible effect of the plastic deformations on the modeled local stiffnesses is evident from the extremely high $r \sim 0.95$ correlation between log-local stiffnesses measured for the prestrained state versus the prestrained relaxed state. Accordingly, there was no substantial difference in the width of the local stiffness distributions for the two cases (inset of Supplementary Fig. 4c). The robustness of our modeled local stiffnesses to persistent lengthening is consistent with our previous results regarding the origin of the variability in local stiffness. That is, our results in the main text demonstrate that the variability of local stiffness is dominated by structural disorder. Since persistent bond-lengthening leaves the network topology unchanged, our main results imply a strong conservation of local stiffness, as indeed we observed. Thus, our results for simulating repeated loading support our conclusion that the heterogeneity of local stiffness is dominated by structural disorder.

Analysis of disordered lattice model

The local stiffness distribution for the FCC lattice network is shown in Supplementary Fig. 5b. Here, we chose a bond-probability of $p = 0.3$ to study the bending regime relevant to biological networks. Throughout our analysis, we set the stretching modulus $\mu = 1$ and bond length $\ell_0 = 1$. We considered local stiffness measurements for pairs of vertices separated by primitive lattice vectors of the form $(-1,\ 2,\ n > 0)$ to avoid pathological effects associated with applying forces in the symmetry directions of the lattice.

For both the SC and FCC disordered lattice networks, we find the presence of a broad range of local stiffnesses spanning up to roughly two decades (Supplementary Figs 11f and 5b). This broad range of stiffnesses is generic over a wide range of network connectivities in the stretching and bending regimes and peaked near the crossovers (see Supplementary Fig. 5c for FCC lattice results). Interestingly, although the range is qualitatively very large, the geometric standard deviation is substantially smaller for both the SC ($\sigma_{\text{loc}} = 0.29$, Supplementary Fig. 11f) and FCC ($\sigma_{\text{loc}} = 0.37$, Supplementary Fig. 5b) lattice networks than for the experimental (collagen: $\sigma_{\text{loc}} = 0.54$, Fig. 2c; fibrin: $\sigma_{\text{loc}} = 0.63$, Supplementary Fig. 1b) and random geometric graph (RGG, $\sigma_{\text{loc}} = 0.56$, Fig. 2d) networks. In the following section, we study how this discrepancy arises from differences in network structure.

What is the physical origin of the qualitatively broad width we observed for the disordered lattice networks? As for the RGG network (Supplementary Fig. 11d-f), the disordered lattice networks also yield macroscopic shear moduli and median stiffnesses that depend sensitively on average coordination number (Supplementary Fig. 11d-f). This suggests that the broad width we observed for all types of networks we studied arises from intrinsic features common to all types of disordered networks. Indeed, as we observed for the RGG network (see Results), we found that for the disordered lattice networks the stiffness loss also obeys universal scaling (see inset of Supplementary Fig. 5b for FCC lattice results), and the scaling of the marginal distribution of local stiffnesses with the number of local fibers N_F is captured by the rapid, nonlinear scaling of the macroscopic shear modulus (insets of Supplementary Fig. 11d-f).

We then estimated the geometric standard deviation for the SC and FCC networks in the same way as for the experimental and RGG networks (see Supplementary Figure 11). As before, we estimate the local stiffness distribution as the distribution of the number of local fibers N_F transformed by the macroscopic shear modulus. For both SC and FCC lattice networks, we found that counting the number of local fibers using rapidly-decaying weighting functions results in a good match between the estimated and actual geometric standard deviations (Supplementary Table 2, Supplementary Fig. 11d-f). Furthermore, we found that the estimate does not depend significantly on the particular

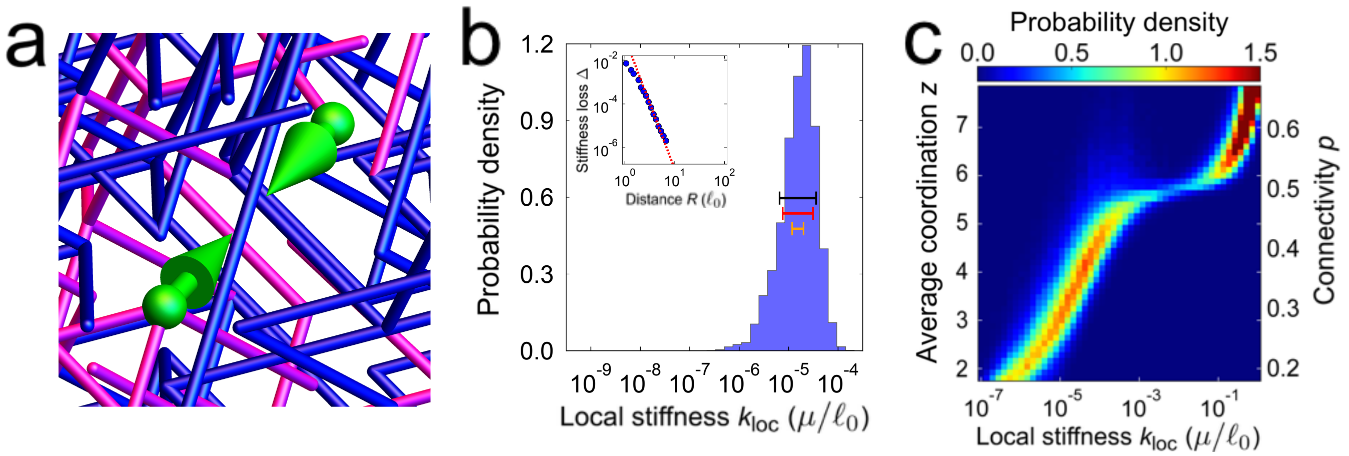

Supplementary Figure 5. Analysis of FCC lattice network. (**a**) Modeled deformation under stress from a local force dipole (green arrows) of disordered face-centered-cubic lattice network (FCC, $d = \sqrt{5}\ell_0$, bond occupancy $p = 0.3$, and bending modulus $\kappa = 10^{-7}\ \mu\ell_0^2$). Magnitude of fiber deformations indicated by color (small deformations blue, large deformations red). (**b**) Distribution of local stiffnesses k_{loc} for FCC network. Geometric standard deviation of local stiffness σ_{loc} indicated by bars (actual distribution black, estimated distribution assuming strong locality red, estimated distribution assuming weak locality orange). Inset shows stiffness loss Δ, defined as the relative change in local stiffness k_{loc} upon perturbing a network by removing a single fiber, versus distance R of center of removed fiber from probe center ($d = \sqrt{5}\ell_0$). Error bars, defined as the standard deviation of each data point divided by the square root of the number of samples averaged, are smaller than the size of data points. Dashed lines show asymptotic scaling from continuum theory, which predicts $\Delta \sim 1/R^{2D}$ for $R >> d$. (**c**) Distribution of local stiffnesses k_{loc} for FCC network versus average coordination number of vertices, z, or equivalently, bond occupancy p (force dipole length $d = \sqrt{5}\ell_0$, bending modulus $\kappa = 10^{-7}\ \mu\ell_0^2$.

form of the weighting function used to calculate the number of local fibers. Specifically, the estimate of the geometric standard deviation for the FCC network does not depend strongly on whether we chose a rapidly decaying power-law or a hard cutoff at two mesh sizes (Supplementary Fig. 11d,e). This suggests that obtaining a good estimate of the geometric standard deviation based on local structure does not require finely-tuning the details of the weighting function, provided the chosen weights are given by a function that decays sufficiently rapidly with distance.

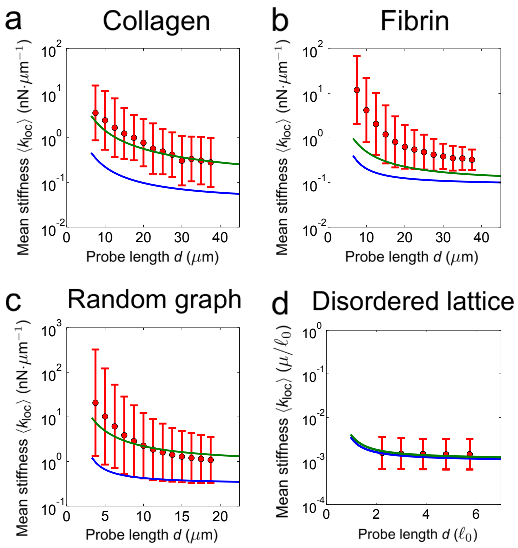

Supplementary Figure 6. Local stiffness versus probe length. (**a-d**) Local stiffness $k_{\rm loc}$ versus probe length d for (**a**) experimental collagen network, (**b**) experimental fibrin network, (**c**) RGG network, and (**d**) FCC network, showing geometric mean $\langle k_{\rm loc}\rangle$ and geometric standard deviation $\sigma_{\rm loc}$ of local stiffness (red); solid curves from conventional continuum elastic theory (blue) and gradient elastic theory (green).

Mean stiffness versus probe length

Above, we demonstrated that the local stiffness distribution is strikingly broad for all types of networks we studied, even under the strict assumption of fixed probe length. Biologically, this distance corresponds to the scale over which cells measure stiffness. Since cells can adopt various sizes and shapes, this means that a cell might measure on different scales depending on the length of its "arms," i.e. the distance between focal adhesions. We therefore consider the local stiffness distribution as a function of the length of the force-dipole probe in our model.

For long probes, the geometric mean of stiffness approaches constant values (Supplementary Fig. 6a-d), as we saw earlier for the geometric standard deviation (insets of Fig. 3a,b). In contrast to the geometric standard deviation, however, the measured stiffness increases by more than a decade as the probe length is decreased to the average mesh size. To understand these features of the mean stiffness as a function of probe length, we developed a simple model for the mean stiffness measured by a local stiffness probe interacting with an isotropic continuum elastic material in three dimensions.

Effective continuum elastic-material model

To gain insight into the average mechanical response of a disordered fiber network to a local force-dipole, we approximate the network as an isotropic continuum elastic material[3]. The response of such an isotropic continuum elastic material can be completely characterized by two macroscopic moduli that appear in the elastic free energy:

$$F = \frac{1}{2}\lambda\epsilon_{ii}\epsilon_{jj} + G\epsilon_{ij}\epsilon_{ij}, \tag{4}$$

where $\epsilon_{ij} = 1/2\left(\partial u_i/\partial x_j + \partial u_j/\partial x_i\right)$ is the strain tensor for the deformation field u_i, λ is the first Lame coefficient, and G is the shear modulus. To determine these elastic parameters of our model, we calculate the shear modulus G and the bulk modulus $K = \lambda + (2/3)G$ of the network.

To compute the shear modulus of a network, we simulate the deformation of a cubical region of network in response to a shear stress. We apply a shear stress via force monopoles applied to all nodes within buffer zones at the top and bottom of the network (see Supplementary Fig. 7a). The deformations of the vertices are obtained by solving the force-balance equation for static equilibrium using SVD (see Methods section of the main text). The shear modulus is defined as

$$G = \frac{F/A}{\bar{\gamma}}, \tag{5}$$

where F is the sum of the magnitudes of all the forces applied to the network, A is the area of the buffer region, and $\bar{\gamma}$ is the average shear strain obtained from the least-squares linear fit of the deformation profile in the direction of the applied force.

To compute the bulk modulus, we simulate the deformation of a spherical region of network in response to a uniform compression. We apply a uniform compression via force-monopoles applied to nodes at the edge of the network (see Supplementary Fig. 7b). The deformations of the vertices are obtained as before by solving the force-balance equation for static equilibrium using SVD. The bulk modulus is defined as

$$B = F/(24\pi R^2 \bar{\epsilon}), \tag{6}$$

where F is the sum of the magnitudes of all the forces applied to the network, R is the distance from the center of the network to the center of the buffer zone, and $\bar{\epsilon}$ is the average radial strain of the nodes within the bulk of the network. Here, the geometric prefactor $\frac{1}{24\pi}$ arises from the spherical geometry.

Continuum stiffness-measuring force-dipole model

The local stiffness probe consists of two force-monopoles separated by a distance $\boldsymbol{d}$ which exert a contractile force of magnitude f_0 (see Supplementary Figure 7c):

$$\boldsymbol{f}(\boldsymbol{r}) = f_0 \left[\delta(\boldsymbol{r}) - \delta(\boldsymbol{r} - \boldsymbol{d})\right] \hat{\boldsymbol{d}}. \tag{7}$$

The deformation produced by each force-monopole is given by the continuum elastic Green's tensor, which is given by the following (in three dimensions)[3]:

$$\mathcal{G}(\boldsymbol{r}_{12}) = \frac{1}{8\pi E}\left(\frac{1+\nu}{1-\nu}\right)\left[(3-4\nu)\mathbb{I} + \boldsymbol{n}\otimes\boldsymbol{n}\right]\frac{1}{r_{12}}, \tag{8}$$

where E is the Young's modulus, ν is the Poisson's ratio, $\mathbb{I}$ is the identity matrix, $\boldsymbol{n}$ is a unit vector parallel to the displacement vector $\boldsymbol{r}_{12}$ between spatial positions 1 and 2, and r_{12} is the magnitude of the displacement vector. The continuum elastic Green's tensor relates applied forces to the resulting deformations of the medium as follows:

$$\boldsymbol{u}(\boldsymbol{r}) = \int \mathcal{G}(\boldsymbol{r}) \cdot \boldsymbol{f}(\boldsymbol{r}) \; dV. \tag{9}$$

Thus, the combination of both force-monopoles acting on the medium results in a deformation $\boldsymbol{u}$ at each point $\boldsymbol{r}$ within the medium:

$$\boldsymbol{u}(\boldsymbol{r}) = f_0 \left[\mathcal{G}(\boldsymbol{r}) - \mathcal{G}(\boldsymbol{r} - \boldsymbol{d})\right] \cdot \hat{\boldsymbol{d}}. \tag{10}$$

We would like to define a local stiffness probe that measures the sum of the deformation u at each monopole along the direction of the probe:

$$u = \boldsymbol{u}(0) \cdot \hat{\boldsymbol{d}} - \boldsymbol{u}(\boldsymbol{d}) \cdot \hat{\boldsymbol{d}} = 2\boldsymbol{u}(0) \cdot \hat{\boldsymbol{d}}, \tag{11}$$

however, in contrast to the network model, here in the continuum model, the force-monopoles induce diverging deformations at the points where the forces are applied.

To account for the unphysical divergences, we define the measured deformation at each point of force application as the average deformation within a spherical cutoff region of radius ξ centered around the point, where ξ is set equal to the mesh size. Intuitively, this choice of cutoff results in a modeled deformation that corresponds to a coarse-grained description of the deformation of a single vertex in the network. The total measured deformation is then given by:

$$\bar{u} = \frac{3}{4\pi\xi^3}\int_{r\leq\xi} u\, d\boldsymbol{r}. \tag{12}$$

We evaluate the integral assuming $d > \xi$ and find the local stiffness measured by the "continuum dipole" is:

$$k_{\text{loc}} = \frac{f_0}{\bar{u}} = \left(\frac{3a_0}{\xi} - \frac{a_0}{d} + \frac{b_0\xi^2}{d^3}\right)^{-1}, \tag{13}$$

where we have defined two constants that depend on the elastic moduli of the network:

$$a_0 = \frac{1+\nu}{\pi E}, \tag{14}$$

$$b_0 = \frac{1}{10\pi E}\left(\frac{1+\nu}{1-\nu}\right). \tag{15}$$

The continuum theory results in monotonically decreasing stiffness as a function of probe length, with stiffness asymptotically approaching a constant value for $d \gg \xi$. This occurs because the measured deformations are produced by two force-monopoles whose deformations add linearly. Since the self-deformation $u_0 = 3a_0/2\xi$ of each monopole remains constant, and the contribution of the far monopole at d decays to zero for $d \gg \xi$, the local stiffness saturates for large probe lengths, i.e. it approaches a constant value determined by the combined deformations of two non-interacting monopoles.

To study the dynamic range of local stiffness as a function of probe length, we consider the fold-stiffening, defined as the ratio $\langle k_{\text{loc}}\rangle/\langle k_\infty\rangle$ of the geometric mean stiffness to the geometric mean stiffness for asymptotically long probes (i.e. $d \to \infty$):

$$\frac{\langle k_{\text{loc}}\rangle}{\langle k_\infty\rangle} = \left(1 - \frac{\xi}{3d} + \frac{b_0\xi^3}{3a_0 d^3}\right)^{-1}. \tag{16}$$

This fold-stiffening predicted from continuum theory is roughly consistent with the fold-stiffening for all types of discrete networks studied when the probe length is at least five times the mesh size (Supplementary Fig. 6). Despite this match in the fold-stiffening at long probe lengths, the observed local stiffness for short probe lengths is always much stiffer than the continuum prediction for the experimental and RGG networks (Supplementary Fig. 6a-c). This discrepancy occurs because the continuum theory does not adequately describe the deformation produced by a monopole at short distances for these networks. This results in a fixed difference between the force-monopole self-deformation predicted by continuum theory and the average deformation produced by a force-monopole applied to the discrete networks.

The effect of long fibers

Below around five times the mesh size, the fold-stiffening measured for the experimental and RGG networks begins to deviate rapidly from the continuum prediction (Supplementary Fig. 6a-c). In contrast, the continuum theory seems to capture the fold-stiffening of the FCC network, which is fairly constant, even down to probe lengths close to the lattice spacing (Supplementary Fig. 6d). What determines the length scale of the extended decay of fold-stiffening we observed for the experimental and RGG networks? In the previous sections, we saw that for fixed probe length, the range of stiffnesses is determined in part by the strong, nonlinear dependence of the marginal distribution of local stiffnesses on network connectivity, with the strongest dependence occurring close to the stretching and bending transitions. Does the scaling of the marginal distribution of local stiffnesses also depend on probe length? If so, the large fold-stiffening we observed for the experimental and RGG networks could occur due to the proximity to an elastic transition.

To determine how network connectivity impacts the fold-stiffening, we varied the average coordination number z for a fixed ratio of the bending modulus κ to the stretching modulus μ. We found that for the RGG network, the dependence of fold-stiffening on probe length becomes much stronger as the average coordination number is tuned to

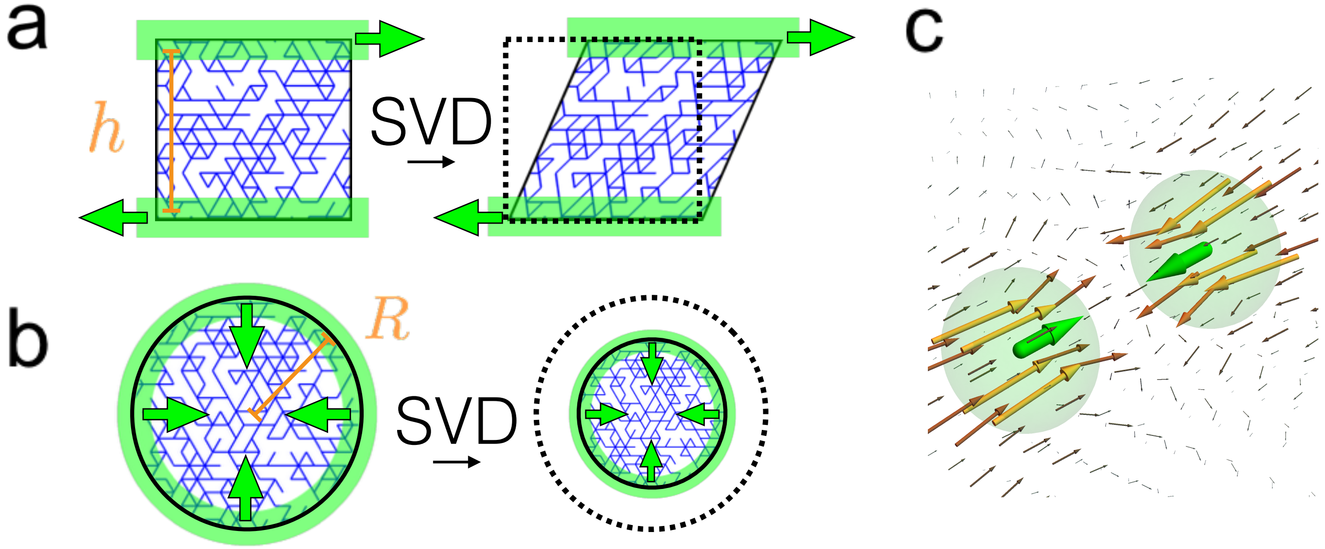

Supplementary Figure 7. Modeling the average stiffness via continuum theory. (**a** and **b**) Schematic illustration of the procedure for computing the elastic moduli used in the continuum theory. (**a**) For the shear modulus, SVD is used to calculate the deformation response to shear forces applied to nodes within buffer zones at the top and bottom of a cubical region of network. The shear modulus is defined as $G = \frac{F/A}{\bar{\gamma}}$, where F is the sum of the magnitudes of all the forces applied to the network, A is the area of the buffer region, and $\bar{\gamma}$ is the average shear strain obtained from the least-squares linear fit of the deformation profile in the direction of the applied force. (**b**) For the bulk modulus, SVD is used to simulate the deformation response to compressive forces applied to nodes within a buffer zone at the boundary of a spherical region of network. The bulk modulus is defined as $B = F/(24\pi R^2\bar{\epsilon})$ where F is the sum of the magnitudes of all the forces applied to the network, R is the distance from the center of the network to the center of the buffer zone, and $\bar{\epsilon}$ is the average radial strain of the nodes within the bulk of the network. (**c**) Schematic illustration of the continuum model for mean stiffness of a network. A local stiffness probe is modeled by two force-monopoles, separated by a distance d, that apply a contractile force to an isotropic continuum elastic material with moduli calculated from the network. The local stiffness k_{loc} in the continuum model is defined as the sum of the average deformation within spheres of radius ξ centered at the points of force application, where ξ is a short-range cutoff equal to the mesh size, defined for the experimental and RGG networks as the radius of a sphere containing the average volume per node, and for the disordered lattice networks as the lattice spacing.

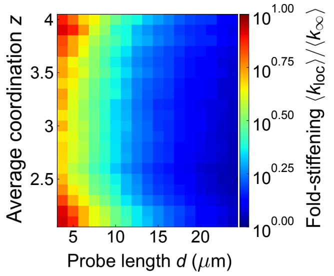

Supplementary Figure 8. Fold-stiffening $\langle k_{\mathrm{loc}}\rangle/\langle k_\infty\rangle$, defined as the geometric mean of local stiffness $\langle k_{\mathrm{loc}}\rangle$ divided by the geometric mean stiffness for asymptotically long probes $\langle k_\infty\rangle$, versus probe length d for RGG network over a range of average coordination number z.

bring the networks closer to an elastic transition (Supplementary Fig. 8). However, even far away from the transitions, there is still a large fold-stiffening at short probe lengths. Since only the experimental and RGG networks have this large fold-stiffening away from the transitions, this property must arise from intrinsic features of the networks that are not present for the FCC network. One feature of the network architecture that explicitly introduces a length scale is the distribution of fiber lengths (Supplementary Fig. 9).

To predict the probe length for which we expect the crossover to short-range stiffening to occur due to the presence of polydisperse fibers, we consider the distribution of fiber lengths for each network. For the disordered lattice networks, we define fiber length as the number of bonds in a filament that are consecutive coaxial segments uninterrupted by a non-coaxial connected segment. We approximate the distribution of fiber lengths $P(\ell)$ as proportional to the

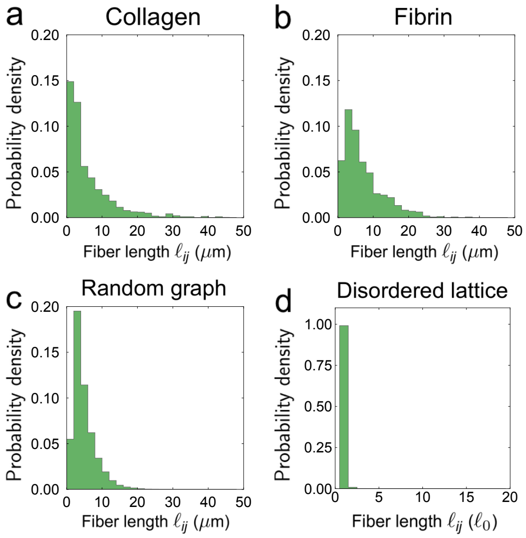

Supplementary Figure 9. (**a**-**d**) Distribution of fiber lengths for (**a**) experimental fibrin network, (**b**) experimental collagen network, (**c**) RGG network, and (**d**) FCC network.

probability of starting from a fiber endpoint and observing ℓ consecutive bonds followed by either a cross-linking (i.e. non-coaxial) bond or the absence of a bond, yielding the following distribution:

$$P(\ell) \propto \left[p(1-p)^{z-2}\right]^{\ell}, \tag{17}$$

where z is the coordination number (6 for the SC lattice and 12 for an FCC lattice). Thus, we find an exponentially decaying distribution with a decay length L given by:

$$L = \frac{-1}{\log\left(p(1-p)^{z-2}\right)}. \tag{18}$$

We see that for the lattice networks, the distribution of fibers decays exponentially. For the FCC network at connectivity $p = 0.3$, the decay length is around $L = 0.2$. This is approximately an order of magnitude shorter than the minimum probe length we considered ($d \gtrsim 2$), which explains why we did not observe the fold-stiffening to deviate from the continuum prediction away from the elastic transitions.

In contrast to the relatively monodisperse fiber-length distribution for the FCC network, the experimental and RGG networks contain long fibers that connect vertices separated by distances that are well beyond the mesh size. Beyond the mesh size ξ, the fiber-length distribution decays roughly exponentially with a length scale approximately equal to the mesh size $L \simeq \xi$. Thus, we expect the long fibers to modify the fold-stiffening out to intermediate probe lengths. Could this effect account for the larger fold-stiffening we observed for the experimental and RGG networks?

The long-ranged structure in the network, e.g. fibers that are several times longer than the probe lengths we considered, precludes us from considering the free energy in the hydrodynamic limit. Thus, to properly describe these networks at these intermediate length scales, we should also consider terms in the free energy that consist, for instance, of gradients of the strain tensor. Although many higher-order terms could contribute at such scales, for simplicity we consider an extension that contains a single additional term[8]. Specifically, for each term in the original elastic energy which contains the squared-sum of the diagonal components of the strain tensor ϵ_{ii} or the squared-sum of the individual components ϵ_{ik}, we also add the vector product of the gradient of each term with itself:

$$\tilde{F} = \frac{1}{2}\lambda\epsilon_{ii}\epsilon_{jj} + G\epsilon_{ij}\epsilon_{ij} + L^2\left(\frac{1}{2}\lambda\epsilon_{ii,k}\epsilon_{jj,k} + G\epsilon_{ij,k}\epsilon_{ij,k}\right), \tag{19}$$

where $\epsilon_{ij,k}$ is the partial derivative of ϵ_{ij} with respect to the coordinate represented by index k, and L is an additional material parameter that corresponds to the extent of the microscopic non-locality. By setting L equal to the decay length obtained from the fiber length distributions of the networks, we show that this gradient elasticity theory

captures the essential features shown in our simulations. The Green's tensor for this non-local continuum theory is given by:

$$\mathcal{G}_{ik}(\boldsymbol{r}) = \frac{1}{32\pi G(1-\nu)}\left[\Psi(r)\delta_{ik} + X(r)n_i n_k\right], \tag{20}$$

where, as in previous work[8], we have defined the two functions

$$\Psi(r) = \frac{2}{r}\left\{(3-4\nu)\left(1-e^{-r/L}\right) + \frac{1}{r^2}\left[2L^2 - (r^2 + 2Lr + 2L^2)e^{-r/L}\right]\right\}, \tag{21}$$

$$X(r) = \frac{2}{r}\left[\left(1 - \frac{6L^2}{r^2}\right) + \left(2 + \frac{6L}{r} + \frac{6L^2}{r^2}\right)e^{-r/L}\right]. \tag{22}$$

Incorporating the additional effect of the strain gradient into the continuum prediction for the experimental and RGG networks results in a fold-stiffening that is approximately a decade at the mesh size ξ, which is significantly larger than the amount predicted by conventional continuum elasticity and captures a majority of the anomalously large fold-stiffening observed for the experimental and RGG networks (Supplementary Fig. 6). In contrast, for the FCC network, the gradient prediction is nearly identical to the conventional continuum prediction, due to the much smaller value of L relative to ξ. These results suggest that accounting for the inherent non-locality from highly polydisperse fibers is crucial to describe the response of biopolymer networks at the cellular scale.

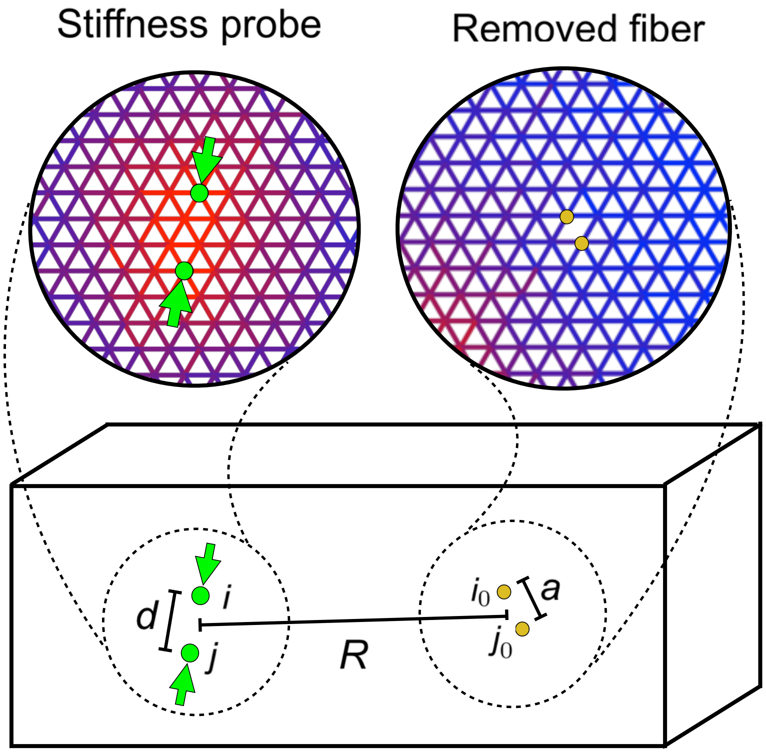

Supplementary Figure 10. Stiffness loss due to fiber removal. Schematic illustration of the geometry used to calculate the fiber-removal effect. The fiber-removal effect is the relative change in local stiffness when a single fiber of length a is removed at a distance $R \gg d$.

Dependence of local stiffness on local structure

To gain insight into the variation of local stiffness, we determined how the presence of a single fiber in the vicinity of the stiffness probe impacts the local stiffness. We quantified the influence of a fiber by the stiffness loss Δ, defined as the relative change in local stiffness upon removing the fiber from the network (Supplementary Fig. 10). Intuitively, fibers which are more proximal should have a greater effect. We therefore expect the stiffness loss induced by removing a fiber to decay as a function of the distance R from the probe center to the center of the fiber. In this section, we calculate how the stiffness loss scales with distance R for an ordered lattice.

Stiffness loss scaling

We calculate the stiffness loss scaling by first deriving an exact formula for the change in local stiffness when a single fiber is removed from an ordered, stretching-only network in D dimensions, and then studying how the magnitude of the effect depends on the distance to the missing fiber from the center of the probe. The equation of force-balance in static equilibrium becomes:

$$\mathcal{D}_0 \cdot \boldsymbol{u} = \boldsymbol{f}, \tag{23}$$

where the unperturbed force-constant matrix $\mathcal{D}_0$, vertex deformations, and applied forces are represented using Dirac notation as follows:

$$\mathcal{D}_0 = \mu \sum_{\langle ij \rangle} |i\rangle \left(b_{ij} \hat{\boldsymbol{r}}_{ij} \otimes \hat{\boldsymbol{r}}_{ij} - \delta_{ij} \sum_{j' \neq i} b_{ij'} \hat{\boldsymbol{r}}_{ij'} \otimes \hat{\boldsymbol{r}}_{ij'} \right) \langle j| \tag{24}$$

$$\boldsymbol{u} = \sum_i |i\rangle \boldsymbol{u}_i \tag{25}$$

$$\boldsymbol{f} = \sum_i |i\rangle \boldsymbol{f}_i, \tag{26}$$

where $b_{ij} = 1$ if vertices i and j are nearest neighbors and $b_{ij} = 0$ otherwise, and the vectors $|i\rangle$ form a complete orthonormal set over vertices such that $\langle i|k\rangle = \delta_{ik}$ and $\sum_i |i\rangle\langle i| = 1$.

These equations can be inverted by Fourier transformation to solve for the displacements:

$$\boldsymbol{u} = \mathcal{G}_0 \cdot \boldsymbol{f}, \tag{27}$$

where $\mathcal{G}_0$ is the lattice Green's tensor defined by $\mathcal{G}_0\mathcal{D}_0 = 1$ and given by

$$\mathcal{G}_0(i,j) \equiv \langle i|\mathcal{G}_0|j\rangle = \int_{k\in BZ} \frac{d^D k}{(2\pi)^D} e^{i\boldsymbol{k}\cdot\boldsymbol{r}_{ij}} \mathcal{D}_0^{-1}(\boldsymbol{k}), \tag{28}$$

in terms of the $D \times D$ dynamical matrix:

$$\mathcal{D}_0(\boldsymbol{k}) = \mu \sum_{\hat{\delta}} \left(1 - e^{i\ell_0 \boldsymbol{k}\cdot\hat{\delta}}\right) \hat{\delta} \otimes \hat{\delta}. \tag{29}$$

Here, $\hat{\delta}$ are unit vectors summed over the directions of the nearest neighbor vertices. The lattice Green's tensor relates the force applied to vertex i to the deformation at vertex j, which provides a simple way to express the relative deformation of the vertices induced by the dipole:

$$u_0 = 2f_0\hat{\boldsymbol{d}} \cdot [\mathcal{G}_0(i,i) - \mathcal{G}_0(i,j)] \cdot \hat{\boldsymbol{d}}. \tag{30}$$

This relative deformation determines the local stiffness defined in Equation 11 of the Methods section of the main text as the ratio of the applied force to the relative deformation $k_{\text{loc}} = f_0/u_0$.

We will now calculate the perturbed stiffness $\tilde{k}_{\text{loc}}$ between vertices i and j when the bond $i_0 j_0$ connecting neighboring vertices i_0 and j_0 is removed, generalizing the analogous calculation for resistor networks[9]. The equation of force-balance for the perturbed network is

$$(\mathcal{D}_0 - \delta\mathcal{D}) \cdot \boldsymbol{u} = \mathcal{D} \cdot \boldsymbol{u} = \boldsymbol{f}, \tag{31}$$

where $\mathcal{D} = \mathcal{D}_0 - \delta\mathcal{D}$ is the force-constant matrix for the perturbed network, i.e. the unperturbed force-constant matrix $\mathcal{D}_0$ minus the contributions associated with the missing bond $i_0 j_0$:

$$\delta\mathcal{D} = \hat{\boldsymbol{r}}_{i_0j_0} \otimes \hat{\boldsymbol{r}}_{i_0j_0} |i_0\rangle\langle j_0| - \hat{\boldsymbol{r}}_{i_0j_0} \otimes \hat{\boldsymbol{r}}_{i_0j_0} |i_0\rangle\langle i_0| + \hat{\boldsymbol{r}}_{j_0i_0} \otimes \hat{\boldsymbol{r}}_{j_0i_0} |j_0\rangle\langle j_0| - \hat{\boldsymbol{r}}_{j_0i_0} \otimes \hat{\boldsymbol{r}}_{j_0i_0} |j_0\rangle\langle i_0| \tag{32}$$

$$= |x\rangle \hat{\boldsymbol{r}}_{i_0j_0} \otimes \hat{\boldsymbol{r}}_{i_0j_0} \langle y|, \tag{33}$$

where $|x\rangle = |i_0\rangle - |j_0\rangle$ and $\langle y| = \langle i_0| - \langle j_0|$. We solve for the deformations as before:

$$\boldsymbol{u} = \mathcal{G} \cdot \boldsymbol{f}, \tag{34}$$

in terms of $\mathcal{G}$, the lattice Green's tensor for the perturbed network defined by $\mathcal{D} \cdot \mathcal{G} = 1$. By premultiplying this equation with $\mathcal{G}_0$, we obtain a recursive formula for $\mathcal{G}$:

$$\mathcal{G} = \mathcal{G}_0 + \mathcal{G}_0 \cdot \delta\mathcal{D} \cdot \mathcal{G}. \tag{35}$$

We substitute the matrix representation of $\delta\mathcal{D}$ given above and iterate to obtain an explicit formula for $\mathcal{G}$ in terms of an infinite series:

$$\mathcal{G} = \mathcal{G}_0 + \left(1 + A + A^2 + \ldots\right) G_0|x\rangle\hat{\boldsymbol{r}}_{i_0j_0} \otimes \hat{\boldsymbol{r}}_{i_0j_0}\langle y|\mathcal{G}_0, \tag{36}$$

where we have defined the constant $A = \hat{\boldsymbol{r}}_{i_0j_0}\langle y|\mathcal{D}_0^{-1}|x\rangle\hat{\boldsymbol{r}}_{i_0j_0}$. We then sum the geometric series to obtain:

$$\mathcal{G} = \mathcal{G}_0 + \frac{\mathcal{G}_0|x\rangle \cdot \hat{\boldsymbol{r}}_{i_0j_0} \otimes \hat{\boldsymbol{r}}_{i_0j_0} \cdot \langle y|\mathcal{G}_0}{1 - \hat{\boldsymbol{r}}_{i_0j_0}\langle y|\mathcal{G}_0|x\rangle\hat{\boldsymbol{r}}_{i_0j_0}}. \tag{37}$$

This is an explicit formula for the lattice Green's tensor of the perturbed network in terms of the lattice Green's tensor of the unperturbed network. The local stiffness measured between vertices i and j is now

$$\tilde{k}_{\text{loc}} = \frac{f_0}{u_0 + \delta u}, \tag{38}$$

with the perturbation δu to the measured deformation given by:

$$\frac{\delta u}{f_0} = \frac{\left[\hat{\boldsymbol{r}}_{ij} \cdot \mathcal{G}_0(i,i_0) \cdot \hat{\boldsymbol{r}}_{i_0j_0} - \hat{\boldsymbol{r}}_{ij} \cdot \mathcal{G}_0(i,j_0) \cdot \hat{\boldsymbol{r}}_{i_0j_0} - \hat{\boldsymbol{r}}_{ij} \cdot \mathcal{G}_0(j,i_0) \cdot \hat{\boldsymbol{r}}_{i_0j_0} + \hat{\boldsymbol{r}}_{ij} \cdot \mathcal{G}_0(j,j_0) \cdot \hat{\boldsymbol{r}}_{i_0j_0}\right]^2}{1 - 2\hat{\boldsymbol{r}}_{i_0j_0} \cdot \left[\mathcal{G}_0(i_0,i_0) - \mathcal{G}_0(i_0,j_0)\right] \cdot \hat{\boldsymbol{r}}_{i_0j_0}}. \tag{39}$$

Using this exact formula for the local stiffness in the vicinity of a single removed fiber, we now approximate the stiffness loss, defined as the relative change in local stiffness upon removing the bond i_0j_0 at a distance R (see Supplementary Fig. 10):

$$\Delta = \left(k_{\text{loc}} - \tilde{k}_{\text{loc}}\right)/k_{\text{loc}}. \tag{40}$$

When the location of the missing fiber is far away from the probe, i.e. $R \gg d$, the perturbation is small and scales as:

$$\Delta \sim \delta u. \tag{41}$$

At these long distances, the lattice Green's tensor approaches the continuum elastic Green's tensor, which is given by the following (in three dimensions)[3]:

$$\mathcal{G}(\boldsymbol{r}_{12}) = \frac{1}{8\pi E}\left(\frac{1+\nu}{1-\nu}\right)\left[(3-4\nu)\mathbb{I} + \boldsymbol{n} \otimes \boldsymbol{n}\right]\frac{1}{r_{12}}, \tag{42}$$

where E is the Young's modulus, ν is the Poisson's ratio, $\mathbb{I}$ is the identity matrix, $\boldsymbol{n}$ is a unit vector parallel to the displacement vector $\boldsymbol{r}_{12}$ between spatial positions 1 and 2, and r_{12} is the magnitude of the displacement vector. For concreteness, in what follows we will restrict our calculation to three dimensions, which will allow us to use the explicit form of the above continuum elastic Green's tensor to approximate how the stiffness loss scales with distance to the missing fiber from the center of the probe. We first note that the denominator of Equation 40 is a constant and can be ignored since we are only interested in how Δ scales. Upon substituting the continuum elastic Green's tensor in place of the lattice Green's tensor, we find that Δ scales as:

$$\Delta \sim \left[\hat{\boldsymbol{r}}_{ij} \cdot \{\mathcal{G}(\boldsymbol{r}_{ii_0}) - \mathcal{G}(\boldsymbol{r}_{ij_0}) - \mathcal{G}(\boldsymbol{r}_{ji_0}) + \mathcal{G}(\boldsymbol{r}_{jj_0})\} \cdot \hat{\boldsymbol{r}}_{i_0j_0}\right]^2 \tag{43}$$

Without loss of generality, we choose coordinates such that the probed vertices lie at positions $i = (-d/2, 0, 0)$ and $j = (d/2, 0, 0)$ and the vertices of the removed fiber lie at positions $i_0 = (R\cos(\psi), R\sin(\psi), 0)$ and $j_0 =$

$i_0 + (a\cos(\theta)\sin(\phi), a\sin(\theta)\sin(\phi), a\cos(\phi))$, where d is the probe length, R is the distance to the removed fiber from the center of the probe, and a is the length of the removed fiber.

Upon substituting the continuum elastic Green's tensor in place of the lattice Green's tensor and expanding the perturbation to the lowest nonvanishing order in $1/R$, we find that the stiffness loss scales as

$$\Delta \sim \frac{a^2 d^2}{E^2}\left(\frac{\nu+1}{\nu-1}\right)^2 \frac{A(\theta,\phi,\psi)}{R^6} \tag{44}$$

where A is a function that depends only on the angles θ, ϕ, and ψ:

$$\begin{aligned} A(\theta,\phi,\psi) = (8 + \cos(2(\theta-\phi)) + 8\cos(2\phi) + \\ \cos(2(\theta+\phi)) + 48\cos(\phi)^2\cos(2\psi) - 2\cos(2\theta)(1 + 30\cos(4\psi)\sin(\phi)^2) + \\ 4\sin(\phi)^2(4(-3+4\nu)\cos(\theta)(\cos(\theta)) + 3\cos(\theta-2\psi)) - 15\sin(2\theta)\sin(4\psi))^2 \end{aligned} \tag{45}$$

We now read off the scaling of stiffness loss with distance R and find:

$$\boxed{\Delta \sim \frac{1}{R^{2D}}} \tag{46}$$

in D dimensions. Although continuum elasticity gives pathological behavior in one dimension, it is straightforward to generalize this result for $D \geq 2$. Comparing this universal result to the average stiffness loss measured for disordered networks (insets of Fig. 2c,d, Supplementary Fig. 1b, and Supplementary Fig. 5b), we find that the observed stiffness loss is consistent with the $1/R^{2D}$ scaling prediction for all types of networks considered.

Numerical estimate of the local stiffness distribution

The rapid decay of stiffness loss suggests that the local stiffness largely depends on a very small number of proximal fibers. To quantify this dependence, we estimate the local stiffness distribution by considering the number of local fibers N_F, given by:

$$N_F = \sum_{\langle ij \rangle} \Delta_{ij}, \tag{47}$$

where Δ is a rapidly decaying function that measures the influence of a fiber based on the distance R_{ij} from the center of the probe to the center of the removed fiber:

$$\Delta_{ij} = \begin{cases} 1 & R_{ij} < \tilde{\xi}, \\ \left(\frac{\tilde{\xi}}{R_{ij}}\right)^x & R > \tilde{\xi}. \end{cases} \tag{48}$$

Here, for simplicity we characterize locality by the strength x of the power-law decay beyond a short-range cutoff $\tilde{\xi}$, which we have introduced to eliminate unphysical divergences. Our introduction of a variable exponent x will allow us to later check how the variance of N_F (and consequently our estimate for the local stiffness distribution) depends on the strength of locality. For strong locality, i.e. equal to the rapid $1/R^6$ decay of stiffness loss Δ in three dimensions, k_{loc} is strongly correlated with N_F for all types of networks studied (insets of Supplementary Figs 11a-c and 12a-c). This correlation suggests that N_F explains much of the variance of local stiffness. However, the geometric mean of the marginal distribution of local stiffnesses $\langle k_{\text{loc}}(N_F)\rangle$ scales rapidly and nonlinearly with N_F. Consequently, the geometric standard deviation of N_F is less than half of that of k_{loc}. This underestimation occurs because fibers have inherently cooperative contributions to the local stiffness that are not captured by the weighted sum in Equation 47, which implicitly assumes that fibers contribute to k_{loc} independently.

To understand how the strength of the collective effects scales with fiber density, we considered the macroscopic shear modulus as a function of average coordination number z, or equivalently $\langle N_F\rangle$ for a fixed density of vertices. We found that the scaling of the shear modulus with z was consistent with the scaling of $\langle k_{\text{loc}}(N_F)\rangle$ with N_F (Supplementary

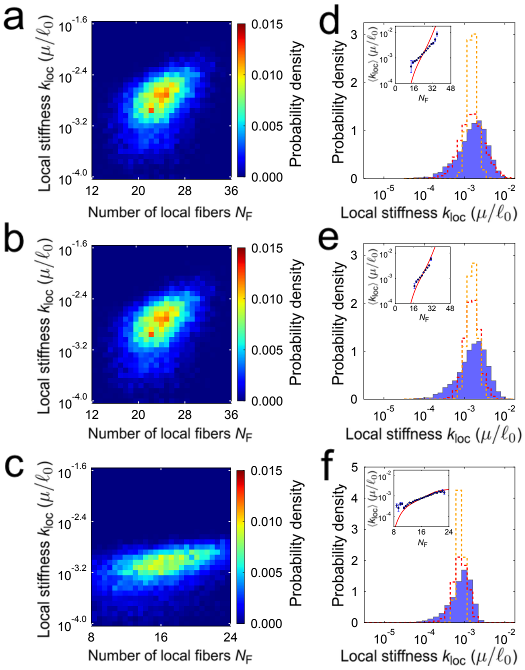

Supplementary Figure 11. Dependence of local stiffness on local structure for disordered lattice networks. (**a-c**) Joint distribution of local stiffness and number of local fibers N_F, defined as a sum of local bonds, for (**a**) FCC network with bond weight $\Delta_{ij} = 1$ below $\tilde{\xi} = \ell_0$ and decaying as $1/R_{ij}^{2D}$ beyond, (**b**) FCC network with bond weight $\Delta_{ij} = 1$ below $\tilde{\xi} = 2\ell_0$ and zero beyond, and (**c**) simple-cubic network with bond weight $\Delta_{ij} = 1$ below $\tilde{\xi} = \ell_0$ and decaying as $1/R_{ij}^{2D}$ beyond. (**d-f**) Distribution of local stiffnesses for (**d**,**e**) FCC network ($d = \sqrt{5}\ell_0$, bond occupancy $p = 0.3$, and bending modulus $\kappa = 10^{-5}\mu\ell_0^2$ where μ is the stretching modulus), and (**f**) simple-cubic network ($d = \sqrt{6}\ell_0$, bond occupancy $p = 0.6$, and bending modulus $\kappa = 10^{-5}\mu\ell_0^2$). Dashed lines show estimated distributions (assuming strong locality as in **a-c** red, weak locality orange, where weak locality is defined by bond weights that decay as $1/R^D$ beyond the cutoff $\tilde{\xi}$ (or zero beyond the hard cutoff $\tilde{\xi} = 4\ell_0$ for **b**). Insets show geometric mean of marginal stiffness distribution $k_{\rm loc}(N_F)$ versus N_F. Error bars are given by the geometric standard deviations divided by the square root of the number of data points; red curves shows the macroscopic shear modulus versus the macroscopic average of the number of local fibers.

Figs 11d-f and 12d-f). We therefore estimated the local stiffness distribution for all types of networks by transforming the numerically-obtained distribution of N_F by the functional dependence of the corresponding shear modulus $G(N_F)$ for a network with average number of fibers equal to $\langle N_F\rangle$, which resulted in a geometric standard deviation given by:

$$\log\sigma_{\rm loc} = \sqrt{\int\left[\log G(N_F) - \langle\log G(N_F)\rangle\right]^2 P(N_F)dN_F}, \tag{49}$$

where

$$\langle\log G(N_F)\rangle = \int \log G(N_F)P(N_F)dN_F. \tag{50}$$

For strong locality, which for concreteness we consider throughout our analysis to be $x = 6$ (equal to the rapid $1/R^{2D}$ scaling of the stiffness loss), the estimator captures the majority of the geometric standard deviation for all types of networks we studied (Supplementary Table 2, Fig. 2c,d, Supplementary Fig. 1b, Supplementary Fig. 5b, and Supplementary Fig. 11f). Furthermore, the estimate does not depend sensitively on the details of the form of the weighting function, as long as the decay is rapid enough (Supplementary Fig. 11d,e). Importantly, this estimator captures the large quantitative discrepancy between the width of the experimental and RGG networks versus that of the lattice networks (Supplementary Table 2). However, for weak locality, which we consider throughout our analysis as $x = 3$, the estimated geometric standard deviation is significantly smaller than the observed value (Fig. 2c,d, Supplementary Fig. 1b, Supplementary Fig. 5b, and Supplementary Fig. 11f).

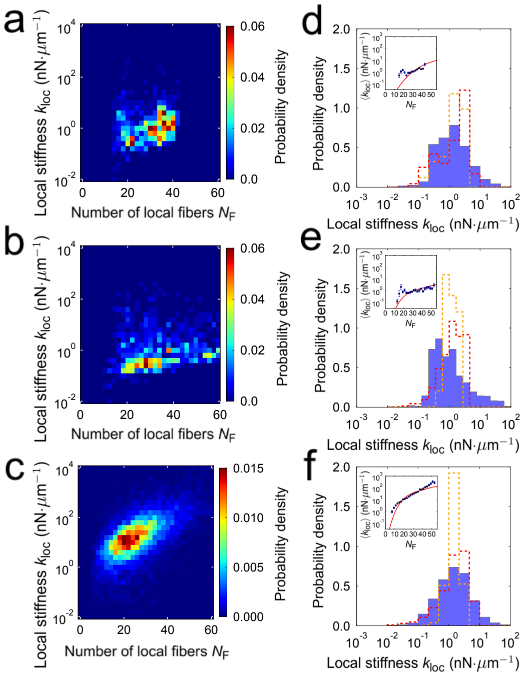

Supplementary Figure 12. Dependence of local stiffness on local structure for experimental and RGG networks. (**a**-**c**) Joint distribution of local stiffness and number of local fibers N_F, defined as a sum of local bonds, for (**A**) collagen network with bond weight $\Delta_{ij} = 1$ below $\tilde{\xi} = 2\xi$ ($\xi \simeq 6.5$ μm for collagen) and decaying as $1/R_{ij}^{2D}$ beyond, (**b**) fibrin network with bond weight $\Delta_{ij} = 1$ below $\tilde{\xi} = 1.5\xi$ ($\xi \simeq 6.7$ μm for fibrin) and decaying as $1/R_{ij}^{2D}$ beyond, and (**c**) RGG network with bond weight $\Delta_{ij} = 1$ below $\tilde{\xi} = 1.5\xi$ ($\xi \simeq 6.7$ μm for RGG) and decaying as $1/R_{ij}^{2D}$ beyond. (**d**-**f**) Distribution of local stiffnesses for (**d**) collagen network network ($d \sim 15\mu$m), (**e**) fibrin network ($d \sim 15\mu$m), and (**f**) RGG network ($d \sim 15\mu$m). Dashed lines show estimated distributions (assuming strong locality as in **a**-**c** red, weak locality orange, where weak locality is defined by bond weights that decay as $1/R^D$ beyond the cutoff $\tilde{\xi}$). Insets show geometric mean of marginal stiffness distribution $k_{\mathrm{loc}}(N_F)$ versus N_F. Error bars are given by the geometric standard deviations divided by the square root of the number of data points; red curves shows the macroscopic shear modulus versus the macroscopic average of the number of local fibers.

Generalized stiffness inference

How accurately can cells infer the *global* stiffness properties of their surroundings based solely on *local* measurements? As we showed above, the global properties are closely related to the overall behavior of the local stiffness distribution. Specifically, the median stiffness tracks the scaling with network connectivity of the macroscopic response. This suggests that a suitable average of several samples of local stiffness could provide useful information about the bulk properties of the network.

To quantify the accuracy of inference, we considered an idealized sampling process that consists of averaging N local stiffness measurements obtained within prolate spheroids. For a given spheroid, the inferred stiffness is given by the geometric mean of N measurements randomly chosen among all the probes whose centers are contained within its volume. We define the relative uncertainty of this estimate as the geometric standard deviation σ_N of the resulting distribution of inferred stiffnesses. This uncertainty is minimized for independent samples, for which σ_N is related to the logarithm of the geometric standard deviation $\log \sigma_{\mathrm{loc}} = \sqrt{\langle(\log k_{\mathrm{loc}} - \langle \log k_{\mathrm{loc}}\rangle)^2\rangle}$ of the distribution of local stiffnesses as follows:

$$\log \sigma_{\mathrm{N}} = \frac{\log \sigma_{\mathrm{loc}}}{\sqrt{N}}. \tag{51}$$

In general, however, stiffness measurements are correlated in space because nearby stiffness measurements depend on shared local structure. To quantify these correlations, we consider the two-point geometric correlation function C_{ij} for local stiffness measurements $k_{\mathrm{loc}}^{(i)}$ and $k_{\mathrm{loc}}^{(j)}$, defined as follows:

$$\log\left(C_{ij}\right) \equiv \frac{\langle(\log k_{\mathrm{loc}}^{(i)} - \langle \log k_{\mathrm{loc}}\rangle)(\log k_{\mathrm{loc}}^{(j)} - \langle \log k_{\mathrm{loc}}\rangle)\rangle}{(\log \sigma_{\mathrm{loc}})^2}. \tag{52}$$

We calculated the two-point geometric correlation function for the experimental and RGG networks and found C_{ij} that were consistent with a decaying exponential form (lower inset of Fig. 6b, inset of Supplementary Fig. 13a):

$$C_{ij} \simeq \frac{C_0 e^{-|\vec{R}_{ij}|/\Lambda}}{(\log \sigma_{\mathrm{loc}})^2}, \tag{53}$$

where $\vec{R}_{ij} = \vec{R}_j - \vec{R}_i$ is the separation between the centers $\vec{R}_i$ of the stiffness probes, Λ is the two-point stiffness correlation length, and C_0 is the zero-distance geometric covariance of local stiffnesses whose centers lie at the same point (C_0 is less than 1 because the centers of distinct probes may coincide). The correlation length Λ and the zero-distance geometric covariance C_0 are obtained by fitting to the modeled local stiffnesses. Due to the presence of these spatial correlations, the uncertainty of inference σ_N will be larger than the uncertainty $\sigma_{\mathrm{loc}}/\sqrt{N}$ predicted by assuming samples of local stiffness are independent. Provided that we can approximate the uncertainty using only the two-point geometric correlation function, the geometric standard deviation $\sigma\left(\left\{\vec{R}_1, \vec{R}_2, \ldots \vec{R}_N\right\}\right)$ (where $\left\{\vec{R}_i\right\}$ is the position of sample i for each of the N samples) is given by:

$$\log \sigma\left(\left\{\vec{R}_i\right\}\right) = \left(1 + \sum_{i<j} \log C_{ij}\right)^{1/2} \frac{\log \sigma_{\mathrm{loc}}}{\sqrt{N}}. \tag{54}$$

For our idealized sampling process, the distances between the samples vary because the probes are positioned randomly throughout the network. In this case, each configuration provides an additive contribution to the variance with a weight proportional to its probability. Since the probes are distributed homogeneously and isotropically throughout space, the overall variance of the inference is simply given by integrating the positions of the N samples over the sampling volume and normalizing by V^N. Upon substituting the form of C_{ij} from above, we find an uncertainty σ_N given by:

$$\log \sigma_{\mathrm{N}} = \left(\frac{(\log \sigma_{\mathrm{loc}})^2}{N} + \frac{N(N-1)C_0}{4V^2} \int_{\vec{R}_1 \in V} \int_{\vec{R}_2 \in V} e^{-|\vec{R}_1 - \vec{R}_2|/\Lambda}\, d\vec{R}_1 d\vec{R}_2\right)^{1/2}. \tag{55}$$

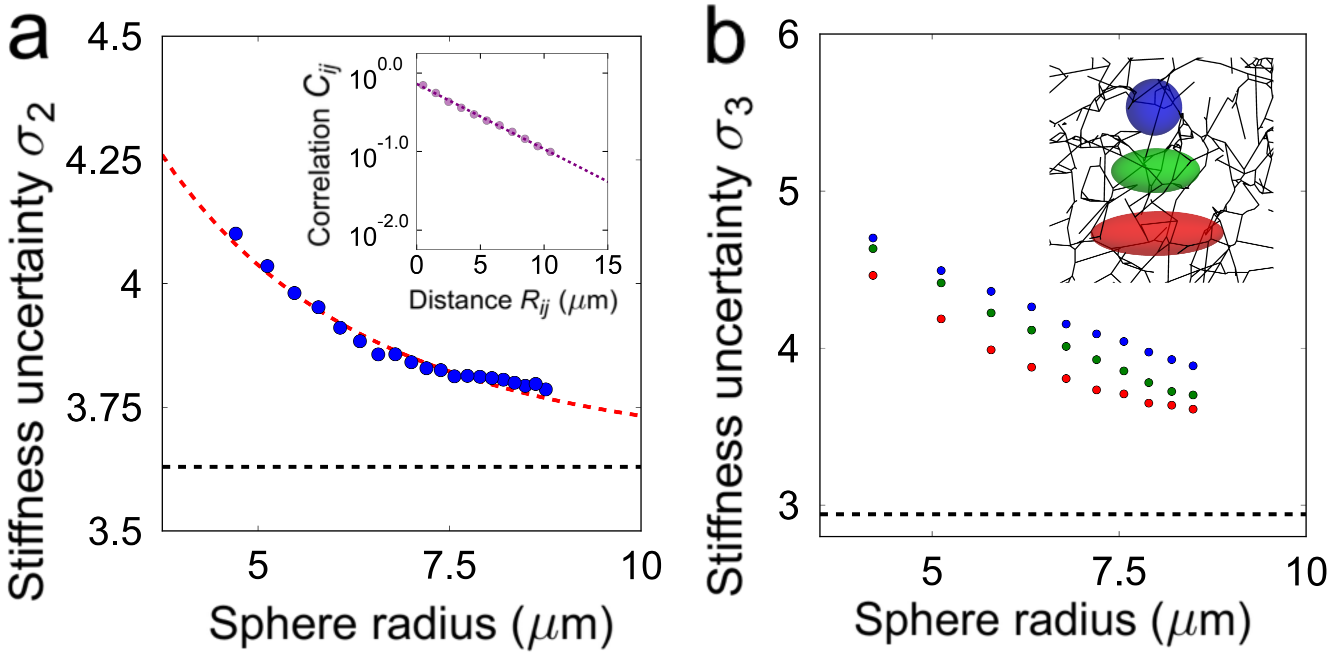

Supplementary Figure 13. Minimum uncertainty of stiffness inference for RGG network. (**a**) Fractional uncertainty of stiffness inference from two measurements, defined as the geometric standard deviation σ_2 of the geometric mean of a random sample of $N = 2$ local stiffnesses measured by force dipoles whose centers lie within spheres versus radius of spheres. Dashed black line shows the geometric standard deviation $\sigma_{\mathrm{loc}}^{1/\sqrt{2}}$ of the geometric mean of $N = 2$ independent measurements, dashed red line shows the uncertainty calculated using the two-point geometric correlation of local stiffnesses. *Inset* shows the two-point geometric correlation ρ_{ij} for local stiffness, defined as the covariance between log-local stiffnesses $\log k_{\mathrm{loc}}^{(i)}$ and $\log k_{\mathrm{loc}}^{(j)}$ divided by the logarithm of the geometric standard deviation of local stiffness squared $(\log \sigma_{\mathrm{loc}})^2$, versus distance R_{ij}. (**b**) Fractional uncertainty of stiffness inference from three measurements, defined as the geometric standard deviation σ_3 of the geometric mean of a random sample of $N = 3$ local stiffnesses measured by force dipoles whose centers lie within spheres (Inset: blue) and prolate spheroids of equivalent volume (Inset: green, aspect ratio 2:1, and red, aspect ratio 3:1) versus radius of spheres. Dashed black line shows the geometric standard deviation $\sigma_{\mathrm{loc}}^{1/\sqrt{3}}$ of the geometric mean of $N = 3$ independent measurements.

For the simple case in which the sampling volume is a sphere of radius R_0, this becomes:

$$\log \sigma_{\mathrm{N}} = \left(\frac{(\log \sigma_{\mathrm{loc}})^2}{N} + N(N-1)C_0 \frac{3\Lambda^3 \left(4R_0^3 - 9R_0^2\Lambda + 15\Lambda^3 - 3e^{-2R_0/\Lambda}(R_0+\Lambda)(2R_0^2 + 5R_0\Lambda + 5\Lambda^2)\right)}{8R_0^6} \right)^{1/2}. \tag{56}$$

We measured the uncertainty σ_2 of the two-sample stiffness inference process for the RGG network and confirmed that it matches the above direct calculation based on the two-point geometric correlation of local stiffnesses of the network (Supplementary Fig. 13a). This consistency demonstrates that the overall uncertainty of inference for sampling two stiffnesses within a volume can be characterized in terms of the underlying two-point correlations among stiffness measurements.

Supplementary Tables

	Collagen	Fibrin (0.2 mg ml^{-1})	Fibrin (0.8 mg ml^{-1})	Fibrin (1.6 mg ml^{-1})
Avg. coordination number z	2.9	2.7	2.8	2.9
Mesh size ξ (μm)	6.5	6.7	4.3	3.0
Avg. fiber length $\langle \ell_{ij} \rangle$ (μm)	6.2	7.1	4.8	3.5
Shear modulus G (Pa)	0.3	1	10	45
Bending modulus κ (nN$\cdot\mu$m^2)	0.38	2.0	3.6	90

Supplementary Table 1: Network properties for all types of experimental networks studied.

	Collagen	Fibrin	RGG	FCC	SC
σ_{loc}	0.54	0.63	0.56	0.37	0.29
σ_{th}	0.49	0.40	0.49	0.30	0.19

Supplementary Table 2: Numerically-simulated and theoretically-estimated geometric standard deviations for all types of networks studied.

Supplementary Discussion

Estimated thermal effects are negligible.

For the analysis performed above, we assumed that the fibers are sufficiently stiff that thermal effects may be neglected. Thermal effects may be safely neglected provided that *i*) the persistence length of fibers is much larger than the mesh size of the network, and *ii*) the enthalpic longitudinal response of a fiber dominates the entropic longitudinal response[4]. To determine whether these assumptions are justified, we first use the fitted bending moduli to estimate the average persistence length ℓ_p as follows:

$$\ell_p = \frac{\kappa}{k_B T}, \tag{57}$$

where k_B is Boltzmann's constant and T is the temperature. For temperatures around those of the human body, our fitted bending moduli yield persistence lengths all around one meter or larger. Since these values are well above the mesh sizes we observed, we expect the fibers to bend like rods on the relevant scales in the ECM.

To determine whether the enthalpic longitudinal response of fiber networks dominates the entropic longitudinal response, we compare the effective mechanical spring constant to the effective thermal spring constant[4]. We expect the enthalpic contribution to dominate if $(a^2\ell_p)^{1/3}$ is larger than the mesh size, where a is the diameter of a fiber. For a persistence length of one meter and a fiber diameter of 100 nm (a typical value for collagen, for example), we find that $(a^2\ell_p)^{1/3}$ is more than 20 μm, which is several times larger than the mesh sizes we observed. Since the longitudinal response is dominated by the enthalpic contribution, we expect fibers to stretch like rods on such scales.

We note that fibers longer than 20 μm (which comprise a small fraction of the networks) may receive entropic contributions to their stretching moduli that are comparable to their enthalpic longitudinal response. For these long fibers, their mechanical response can still be described as two mechanical springs in series, albeit with a nonlinear compliance. Since we expect the stiffnesses of these two springs to be comparable, the fibers should still be much softer to bending than to stretching. We therefore expect that our main results, regarding bending-dominated networks, will be unaffected by these small corrections. Taken together, these estimates for the bending and stretching of fibers in the ECM suggest that thermal effects are negligible and that the mechanical response is dominated by fiber elasticity.

Supplementary Methods

Modeled network architectures

To understand how the mechanical response emerges from the features of the network architecture, we considered idealized model architectures. For simplicity, the resulting structures were generated randomly such that the initial positions of the vertices and fibers were not spatially correlated. Throughout our analysis, we obtained our results for these networks by averaging over a large number of network realizations to reduce sampling noise.

Random geometric graph model

Random geometric graphs (RGG) provide a way to generate model architectures with independent control over average coordination number, vertex density, and average fiber length. In particular, these parameters can be chosen to match those observed in experiment. In the RGG model, architectures are generated by first distributing vertices throughout a volume and then connecting them randomly according to a specified probability distribution. The position of each vertex is drawn from a uniform distribution over the network volume, and the probability distribution for connecting two vertices, or the "connection function," depends only on the inter-vertex distance. For simplicity, we model the connection function as a power law with an exponential cutoff:

$$P_c(\ell) \propto \frac{e^{-\ell/L}}{\ell^2}, \tag{58}$$

where L is a length scale that describes the typical length of fibers. This results in networks that are homogeneous and isotropic over large scales. For such networks in three dimensions, the expected density of vertices that are a distance ℓ from a particular vertex scales as ℓ^2, so the expected distribution of fiber lengths in the three-dimensional RGG model is $P(\ell) \propto \ell^2 P_c(\ell) \propto e^{-\ell/L}$.

Throughout our analysis, we considered networks with approximately $6.25 \cdot 10^{-3}$ vertices/μm^3 and we chose the length scale L equal to 3.5 μm. We chose the average coordination number $z \simeq 2.9$ to match that of the experimental networks (except in Fig. 2a where we varied the average coordination number by randomly removing fibers from a network with a high initial average coordination number). This resulted in an average mesh size $\xi \simeq 6.7$ μm. Due to the larger resulting density of these modeled networks in comparison to the experimental networks, we considered samples of radius $R_{\text{sample}} = 33$ μm. We determined the bending modulus κ by fitting the geometric mean stiffness measured for the RGG network at $d = 15$ μm to that of the collagen network. This resulted in a bending modulus $\kappa = 83$ nN$\cdot\mu$m^2 and shear modulus $G = 9$ Pa. For the above choices of parameters, the RGG networks quantitatively capture the local mechanical response of the experimental networks, including the broad width of the local stiffness distribution, the large fold-stiffening for short probes, and the two-point stiffness correlation length (see Results). Because of the broad range of stiffnesses, throughout our work, we characterize the variability of the stiffness distribution using the geometric standard deviation, defined as the exponential of the standard deviation of log-local stiffnesses:

$$\sigma_{\text{loc}} = e^{\sqrt{\langle(\log k_{\text{loc}} - \langle \log k_{\text{loc}}\rangle)^2\rangle}} \tag{59}$$

Disordered lattice model

Disordered lattice networks are an established class of modeled architectures that have successfully described many of the mechanical properties of biopolymer networks[4]. In particular, lattice models are able to capture the macroscopic response and its striking dependence on network density. Here, we provide the first numerical analysis of the microscopic response of three-dimensional disordered lattice networks and demonstrate that such models fail to quantitatively capture the broad width we observed for the experimental collagen and fibrin networks.

Disordered lattice networks are generated by placing vertices on an ordered lattice, for example face-centered-cubic (FCC) or simple-cubic (SC) lattices in three dimensions, and connecting them randomly with a fixed probability p. For a given choice of lattice, the connectivity p is the sole parameter that impacts the overall network structure. In particular, the density of vertices cannot be varied independently from the average fiber length. Furthermore, since the vertices are initially positioned to lie on a regular lattice, fibers only meet at discrete, fixed angles in the unperturbed reference state. Disordered lattice networks are therefore significantly simpler than the experimental networks.

Nonlinear fiber network model

The mechanical energy defined in the Methods section of the main text is fully linear in the deformations $\boldsymbol{u}_i$ and therefore does not account for geometrical nonlinearities that occur due to rearrangement of fibers. Therefore, to study networks that may be highly deformed by prestress, we also considered the following mechanical energy:

$$H = \frac{\mu}{2} \sum_{\langle ij \rangle} \frac{\left(|\boldsymbol{x}_{ij}| - \ell_{ij} \right)^2}{\ell_{ij}} + \frac{\kappa}{2} \sum_{\langle ijk \rangle} \frac{\left(\theta_{ijk} - \theta_{ijk}^{(0)} \right)^2}{\ell_{ijk}}, \tag{60}$$

where μ is the stretching modulus, κ is the bending modulus,

$$\boldsymbol{x}_{ij} = \boldsymbol{x}_j - \boldsymbol{x}_i \tag{61}$$

is the difference in positions of vertices i and j, ℓ_{ij} is the length of the fiber ij connecting vertices i and j in the unperturbed reference state (i.e. the unstretched fiber length),

$$\ell_{ijk} = \left(\ell_{ij} + \ell_{jk} \right) / 2 \tag{62}$$

is the average unstretched length of fibers ij and jk,

$$\theta_{ijk} = \sin^{-1} \left(|\hat{\boldsymbol{x}}_{ij} \times \hat{\boldsymbol{x}}_{jk}| \right) \tag{63}$$

is the angle between fibers ij and jk that meet at vertex j,

$$\theta_{ijk}^{(0)} = \sin^{-1} \left(|\hat{\boldsymbol{r}}_{ij} \times \hat{\boldsymbol{r}}_{jk}| \right) \tag{64}$$

is the angle between unit vectors $\hat{\boldsymbol{r}}_{ij}$ and $\hat{\boldsymbol{r}}_{jk}$ that point along the direction of the fibers connecting vertices in the unperturbed reference state. As before for the linear Hamiltonian, the first sum corresponds to stretching interactions and is taken over all fibers ij. The second sum constrains angular deflections of all connected pairs of fibers ij and jk. Although all terms of the sums are Hookean springs and torsional springs, the overall response is subject to geometrical nonlinearities due to rearrangement of fiber segments.

Supplementary References

[1] Reese, S.P., Farhang, N., Poulson, R., Parkman, G., & Weiss, J.A. *Nanoscale Imaging of Collagen Gels with Focused Ion Beam Milling and Scanning Electron Microscopy. Biophys J* **111,** 1797-1804 (2016).

[2] Shah, G.A., Ferguson, I.A., Dhall, T.Z., & Dhall, D.P. *Polydispersion in the Diameter of Fibers in Fibrin Networks: Consequences on the Measurement of Mass-Length Ratio by Permeability and Turbidity. Biopolymers* **21,** 1037-1047 (1982).

[3] Landau, L.D., Lifshitz, E.M., Kosevich, A.M. & Pitaevskii, L.P. *Theory of Elasticity. Pergamon Press, London* (1986).

[4] Broedersz, C.P. & MacKintosh, F.C. *Modeling semiflexible polymer networks. Rev Mod Phys* **86,** 995 (2014).

[5] Huisman, E.M., Storm, C., & Barkema, G.T. *Monte Carlo study of multiply crosslinked semiflexible polymer networks. Phys Rev E* **78,** 051801 (2008).

[6] Das, M., Quint, D. & Schwarz, J.M. *Redundancy and cooperativity in the mechanics of compositely crosslinked filamentous networks PLoS One* **7,** 35939 (2012).

[7] Rens, R., Vahabi, M., Licup, A.J., MacKintosh, F.C., & Sharma, A. *Nonlinear Mechanics of Athermal Branched Biopolymer Networks. J Phys Chem B* **120,** 5831-5841 (2016).

[8] Gao, X.L. & Ma, H.M. *Green's function and Eshely's tensor based on a simplified strain gradient elasticity theory. Acta Mech* **207,** 163-181 (2009).

[9] Cserti, J., David, G. & Piroth, A. *Perturbation of infinite networks of resistors. Am J Phys* **70,** 153 (2002).
